# Observation and Analysis of Staircase Response of Single Palladium Nanoparticle Collision on Gold Ultramicroelectrodes

**DOI:** 10.3390/nano12183095

**Published:** 2022-09-07

**Authors:** Hubert Rudakemwa, Ki Jun Kim, Tae Eun Park, Hyeryeon Son, Jaedo Na, Seong Jung Kwon

**Affiliations:** Department of Chemistry, Konkuk University, 120 Neungdong-ro, Gwangjin-gu, Seoul 05029, Korea

**Keywords:** palladium, single nanoparticle, collision, electrocatalytic amplification, staircase response

## Abstract

Collision (or impact) of single palladium nanoparticles (Pd NPs) on gold (Au), copper (Cu), nickel (Ni), and platinum (Pt) ultramicroelectrodes (UMEs) were investigated via electrocatalytic amplification method. Unlike the blip responses of previous Pd NP collision studies, the staircase current response was obtained with the Au UME. The current response, including collision frequency and peak magnitude, was analyzed depending on the material of the UME and the applied potential. Adsorption factors implying the interaction between the Pd NP and the UMEs are suggested based on the experimental results.

## 1. Introduction

Palladium (Pd) nanoparticles (NPs) have been used as an electrocatalyst for various applications such as energy conversion, storage, and other fields [1,2,3,4]. NPs demonstrate various catalytic activity depending on their atomic morphology and nano dimension [5,6]. Therefore, the characteristic investigation of NPs at a single-NP level is important for its applications. However, since the catalytic current generated by a single NP is tiny, it is difficult to observe and analyze the signal.

The electrochemical amplification (EA) method has been studied by many groups [7,8,9,10,11,12,13,14,15,16]. This method is suitable for the investigation of electrocatalytic activity of NPs at a single-NP level in situ because it can measure the single NP’s electrocatalytic response when the single NP collides on the small electrode such as an ultramicroelectrode (UME). Metal NPs such as Pt [8,9], Au [10,11], and Ag [12,13] have been widely studied with the EA method, but Pd NPs have rarely been studied despite having a significant electrocatalytic activity. Previously, Zare’s group investigated the electrochemical activity of a single Pd NP for hydrazine oxidation using a carbon fiber (C fiber) UME [17]. We investigated the activity of a Pd NP for the hydrogen reduction using a Au UME [18]. In both studies, the current responses of the Pd NP were obtained only with blip (or spike) responses. The blip (or spike) response is obtained when the collided NP leaves the electrode surface or is deactivated by some reason such as impurity adsorption or metal alloying between the NP and the UME [7,19]. Therefore, the blip response of a Pd NP seems to be due to the low adsorption property between the Pd NP and UMEs or the loss of activity of the Pd NP. In contrast, the staircase current response of an NP is obtained when an NP collides and maintains its catalytic activity [8]. Since the staircase response is based on a steady-state current, it is more useful to analyze its magnitude and frequency by a theoretical approach compared to the blip response.

In this study, the electrocatalytic activity of a Pd NP for hydrazine oxidation was investigated using the EA method with various electrode materials, such as copper (Cu), nickel (Ni), platinum (Pt), and gold (Au) to find out the best supporting electrode material. At the Au UMEs, the staircase response was observed with 0.1 V (vs. Ag/AgCl) of applied potential. The peak height and frequency of the staircase responses were analyzed and compared with each other. In the EA method, the type of current response by the single NP’s collision onto the UME is affected by various factors including the material of the UME [19,20]. The staircase and blip responses of the current signal indicate that the different behaviors of NPs occur when they collide. Therefore, the analysis of current response can provide insights about the selection of a supporting electrode. [9] This is important for the application of NPs, because it affects the electrical conductivity or electron transfer between the NPs and the electrode.

## 2. Materials and Methods

### 2.1. Reagent

Palladium (II) chloride (PdCl_2_, 99%), sodium borohydride (NaBH_4_, ≥96%), and citric acid were obtained from Sigma or Aldrich (St. Louis, MO, USA). All chemicals were used as received. Ultrapure water (≥18 MΩ, Millipore, Burlington, MA, USA) was used in all experiments. Pd and Ni wires were obtained from Goodfellow (Devon, PA, USA). Pt, Au, and Cu wires were obtained from Alfa Aesar (Ward Hill, MA, USA).

### 2.2. Preparation of Pd NP

**Figure 1:**
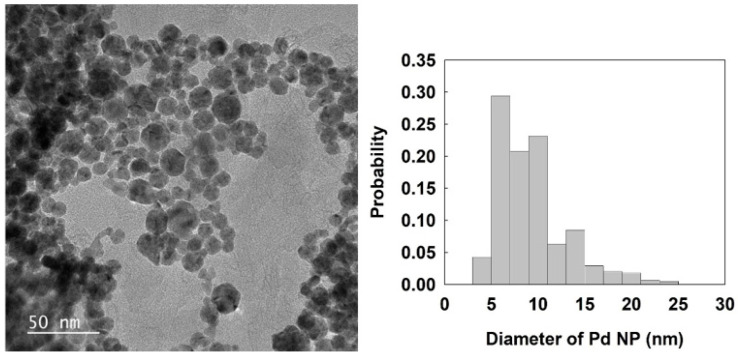
TEM image and size distribution of Pd NPs. The scan bar is 50 nm. The average size and standard deviation of Pd NPs were 9.3 (±3.8) nm.

PdCl_2_ was reduced by NaBH_4_ in the presence of sodium citrate [21]. In this experiment, 1.77 mg of PdCl_2_ (10 µmol) was dissolved in 20 mL of 1 mM HCl under ultrasonic treatment for 30 min. Then, 3.84 mg of citric acid (20 µmol) was added in the solution at 80 °C. After that, 2 mL of 0.10 M NaBH_4_ was slowly added to the mixture under vigorous stirring. The size of the Pd NPs was estimated from the transmission electron microscopy (TEM) measurement. The obtained average diameter and standard deviation of Pd NP were 9.3 nm (±3.8), as shown in Figure 1.

### 2.3. Preparation of UME

We used the following process to prepare Pd (20 μm of diameter), Pt (25 μm of diameter), Au (25 μm of diameter), Cu (15 μm of diameter), and Ni (25 μm of diameter) UMEs [7,8]. Briefly, a metal wire was inserted into a one-side sealed capillary tube. The other side of the tube was connected to a vacuum pump. Then, the sealed end of a glass was heated by a red-hot nichrome coil under vacuuming until the metal wire was fixed to the tube. Next, the opposite end of the metal wire was connected to a lead-out wire using a silver epoxy. The tube was ground with sandpaper to expose the metal phase and polished with alumina powder (0.05 μm) on microcloth pads (Buehler, Lake Bluff, IL) to obtain a mirror-like surface. The projected surface area and the quality of UMEs were checked by the voltammetry of the ferrocene methanol oxidation in an aqueous solution.

### 2.4. Instrumentation

The electrochemical measurement was performed using a CHInstruments model 660D potentiostat (Austin, TX, USA) with the three-electrode cell system placed in a Faraday cage. A Pt wire and a Ag/AgCl (1 M KCl) electrode were used as the counter and the reference electrode, respectively. All potentials are reported vs. Ag/AgCl. TEM images were obtained from the National Center for Inter-University Facilities (NCIRF, Seoul, Korea) using JEM-F200 (JEOL, Tokyo, Japan). The solution of the electrochemical cell contained 50 mM of a phosphate buffer (PB, pH 6.8) with 15 mM of hydrazine. The 50 mM of electrolyte concentration was optimized by many experiments in the single-NP collision experiment (Figure S1, see the Supporting Information, SI). When the concentration of electrolyte is too low, the solution resistance increases and the buffer capacity decreases, thus the observation of the current signal is difficult. If the concentration of electrolyte is high, the aggregation of NPs is acerated.

## 3. Results and Discussion

The electrocatalytic amplification method to observe single-nanoparticle (NP) collisions is based on the different electrocatalytic ability between an NP and an ultramicroelectrode (UME) [7,8]. Usually, the NP and UME are prepared with the better and less electrocatalytic materials for a certain electrocatalytic reaction, respectively. For the effective observation of a single-NP collision signal, the electrocatalytic current should be obtained only by the NP collided on the UME. Therefore, the selection of the appropriate applied potential to the UME is important to obtain an observable current signal. When an excessive potential is applied, the electrocatalytic reaction by the NP is vigorous and the current step by the NP is increased. However, at the same time, the background current from the UME is increased, too. The increased background current affects the sensitivity in signal detection. Therefore, the potential that maximizes the difference of electrocatalytic current between the NP and the UME has to be selected for the effective detection of single-NP collisions.

**Figure 2:**
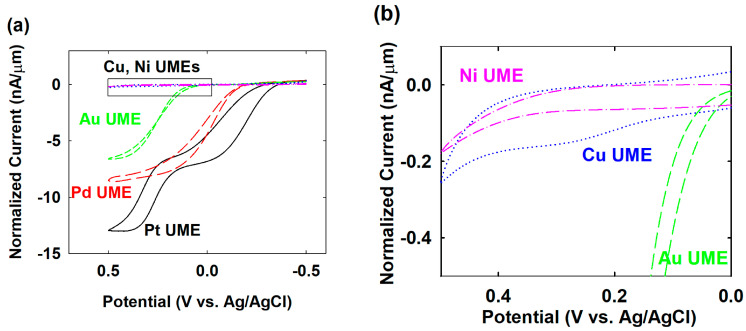
(**a**) Cyclic voltammograms of hydrazine oxidation on Pd (red long-dashed, diameter 20 µm), Pt (black solid, diameter 25 µm), Au (green dashed, diameter 25 µm), Ni (pink dash-dotted, diameter 25 µm), and Cu (blue dotted, diameter 15 µm) UME in a 50 mM phosphate buffer (pH 6.8) containing 15 mM hydrazine. Currents are normalized for the radius of each UME. (**b**) Zoomed-in insert of (**a**).

To find the available potential for palladium (Pd) NPs’ collision with various electrode materials, such as copper (Cu), nickel (Ni), gold (Au), and platinum (Pt), the electrocatalytic activity for hydrazine oxidation was investigated using Pd, Cu, Ni, Au, and Pt UMEs. The behavior of the hydrazine oxidation of the Cu, Ni, Au, and Pt UMEs differed from that of a Pd UME, as shown in Figure 2. In order to facilitate the comparison on the hydrazine oxidation, the normalized current obtained by dividing the raw current by the radius of each UME was used. Not only the Pd but also the Au and Pt UMEs had considerable electrocatalytic activity for the hydrazine oxidation. The onset potential at Pt, Pd, and Au UMEs were −0.4, −0.2, and 0.1 V (vs. Ag/AgCl), respectively. The Pt UME showed similar or better electrocatalytic activity than that of the Pd UME. As such, if the difference of electrocatalytic activity between Pt and Pd was not large enough, the Pt UME was not suitable for the single-Pd-NP collision experiments, theoretically. However, practically, some combination of NP and UME, for example Pt NP-casted Pd UME, showed a synergetic catalytic current similar to that of NP or UME alone [22]. In this work, the Pd NP-collided Pt UME also showed this kind of enhanced electrocatalytic activity, therefore the Pd NP collision was detectable using a Pt UME. For the Au UME, a specific potential region, for example from −0.2 to 0.1 V, was appropriate for the effective observation of the single-Pd-NP collision. On the other hand, the Cu and Ni UMEs did not significantly electrooxidize the hydrazine in the entire experimental potential region from −0.5 to 0.5 V. However, there was some minor oxidation current rising near 0.2 V at the Cu UME, as shown in Figure 2b, caused by the electrooxidation of the Cu itself [20,23]. As a result of the low electrocatalytic activity of Cu and Ni for the hydrazine oxidation, it was expected that these electrodes would have a wide potential range for each single-Pd-NP collision.

**Figure 3:**
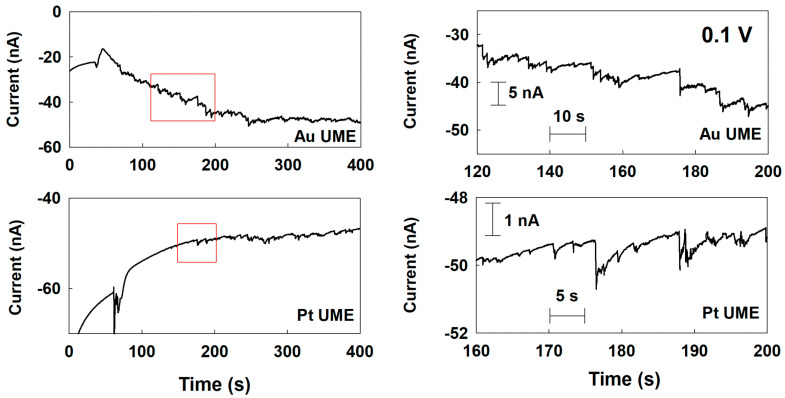
Chronoamperometric curves for a single-Pd-NP collision at the Au and Pt UMEs with a potential of 0.1 V (vs. Ag/AgCl) applied in a 50 mM phosphate buffer (pH 6.8) containing 15 mM hydrazine. The Pd NP concentration was 160 pM. The data acquisition time was 50 ms.

Based on the cyclic voltammetry (CV) measurement, a potential of 0.1 V was firstly applied to the UMEs to obtain the maximum current difference between each electrode material and Pd for the chronoamperometry (CA) measurement. Chronoamperometric curves for single-Pd-NP collisions delivered on the various UMEs at 0.1 V. First, the background noise current in the absence of Pd NP was recorded (Figures S2 and S3). Later, the single-NP collision was investigated in the present of Pd NP. As shown in Figure S4, at the Cu and Ni UMEs, the size of the current response was tiny, and the collision frequency was rare; therefore, it was difficult to distinguish from the background current. Considering the size and frequency, they were not from the typical current response of an NP which was collided and stuck on the UMEs. The tiny and rare responses were probably due to the weak interaction between the Pd NP and those UMEs. According to the Pourbaix diagram, Cu and Ni oxides are the preferred states at this potential and pH [24,25]. Therefore, the Cu and Ni oxide layers on the UME surface may interact differently with the NP or bring additional resistance, thereby affecting the NP collision signal. On the other hand, at the Au and Pt UMEs, typical and clearly distinguishable staircase and blip current responses were observed, respectively (Figure 3). The current steps were at the sub-nanoampere level, much higher than those of Cu and Ni UMEs. In the case of the Pt UME, even though the electrocatalytic activity of Pt is similar to that of Pd at 0.1 V, the collision signal of the single NPs was obtained due to the synergistic effect mentioned earlier [22].

The current response of the single-NP collision could be changed depending on the applied potential to the UME. The change of response was related to the change of reaction mechanism. Previously, when the applied potential was higher, the change from a staircase to a blip response was reported in the Pt NP/Ni UME/hydrazine oxidation system [9]. That was caused by the side effect (nanobubble enclosure) of the reaction product [26,27]. In the case of the Ag NP/Au UME/hydrazine oxidation system, the shape of the signal changed from a reverse staircase to a reverse-blip to a blip response depending on the applied potential, due to the change of state of the Ag NP according to the oxidation progress of the Ag NP [28].

**Figure 4:**
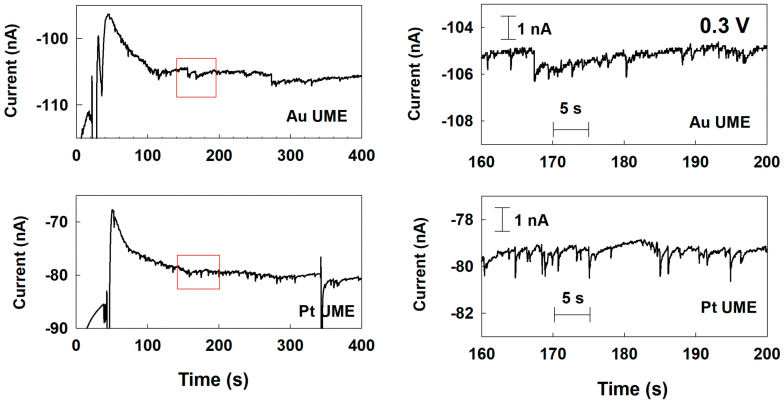
Chronoamperometric curves for a single-Pd-NP collision at Au and Pt UMEs with a potential of 0.3 V (vs. Ag/AgCl) applied in a 50 mM phosphate buffer (pH 6.8) containing 15 mM hydrazine. The Pd NP concentration was 160 pM. The data acquisition time was 50 ms.

Therefore, to identify the change of electrocatalytic reaction of the Pd NP at various UMEs depending on the applied potential, the single-Pd-NP collision was also investigated with a potential of 0.3 V applied to the UMEs. As shown in Figure S5, in the case of Cu and Ni UMEs, there was no current response at all. Because of the increase background current level at 0.3 V comparing to 0.1 V, the small signal could be buried in the background noise. In the Au and Pt UME cases, the blip current transient was observed at 0.3 V (Figure 4). Comparing to the staircase response at 0.1 V in Figure 3, the current response of the Au UME looked less like a staircase, with more noticeable blip-like features. The current response seen at 0.3 V for the Pt UME showed more blip features than the one observed at 0.1 V as well. This change from a staircase to a blip response may be due to the deactivation of the Pd NP, like the Pt NP’s behavior for the hydrazine oxidation in a previous study [9].

As demonstrated in Figure 3 and Figure 4, the change in current response according to the applied potential were due to the change in reaction mechanism, as in the Pt NP/ Ni UME/hydrazine oxidation system mentioned above, or an increase of the background current level of the UMEs. These results showed which electrode material and applied potential were suitable for observing a single-NP collision signal. The observed response types of the collision signals at 0.1 V and 0.3 V are summarized in Table 1.

The peak height and collision frequencies were analyzed to obtain insights on the collision mechanism between the NP and the UMEs. In typical single-NP collision experiments, the peak height of the staircase response indicates the size of the struck NP on the UME. Based on the size of the Pd NP estimated by the TEM image, the theoretical steady-state current value of the Pd NP can be calculated by the following equation: [7,8,29]

(1) $$I_{\mathrm{SS},\mathrm{NP}}=4\pi \left(ln2\right)nFD_{N_{2}H_{4}}C_{N_{2}H_{4}}r_{\mathrm{NP}}$$

where *n* = 4 is the number of electrons for the hydrazine oxidation, *F* is the Faraday coefficient, $D_{N_{2}H_{4}}\;$ is the diffusion coefficient of hydrazine, $C_{N_{2}H_{4}}$ is the concentration of hydrazine, and $r_{\mathrm{NP}}$ is the radius of the Pd NP. Here, the diffusion coefficient of hydrazine is estimated using the steady-state current of the Pd UME in Figure 2 by the following equation: [29,30]

(2) $$I_{\mathrm{SS},\mathrm{UME}}=4nFD_{N_{2}H_{4}}C_{N_{2}H_{4}}r_{\mathrm{UME}}$$

where $I_{\mathrm{SS},\mathrm{UME}}$ is the steady-state current of the Pd UME which is experimentally obtainable, $r_{\mathrm{UME}}$ is the radius of the Pd UME. The diffusion coefficient, 3.24 × 10^−5^ cm^2^ s^−1^, was obtained from Figure 2 using a steady-state current of 75 nA at a 15 mM concentration of hydrazine, a 10 µm radius for the Pd UME, and a four-electron transfer reaction. As a result of the above calculation, the theoretical steady-state current by a single Pd NP was 76 pA. The experimentally obtained current steps for the staircase response and peak height for the blip response were 700 and 325 pA for Au and Pt UMEs, respectively. Even though the size of the Pd NP was the same for all experiments, a different magnitude of the current response was obtained at each UME. The experimental value was higher than the theoretical expectation. The higher value indicated the aggregation of the Pd NP. [29] In addition, the difference between the peak height may indicate the relative strength of the interaction between the Pd NP and each UME. If the collided NPs are strongly stuck on the UME, it could provide a stable steady-state current. Therefore, the relatively smaller peak height for the Cu and Ni UMEs may imply that those UME materials are not adhesive for the Pd NP collisions. In the case of Cu or Ni, oxides are likely to be formed on the electrode surface. Therefore, one probable explanation for this result is that the lattice properties (atom radius and crystal structure) of Au (144 pm, face-centered cubic (fcc)) or Pt (139 pm, fcc) materials may be more similar to those of Pd (137 pm, fcc) materials than the Cu oxide (128 pm, cuprite) and Ni oxide (124 pm, rock salt) materials, allowing their UMEs and NPs to interact more strongly.

The collision frequencies were also investigated at the UMEs. As listed in Table 1, the experimentally obtained normalized collision frequencies for the Au and Pt UMEs were 0.051 and 0.028 s^−1^ μm^−1^, respectively. However, the expected normalized collision frequency, 20.0 s^−1^ μm^−1^, was calculated by the Fick’s law: [29,30]

(3) $$f_{p}=4k_{\mathrm{ads}}D_{\mathrm{NP}}C_{\mathrm{NP}}r_{\mathrm{UME}}$$

where *k*_ads_ is a suggested factor indicating the adsorption properties between the Pd NP and the UME, *C*_NP_ is the concentration of NPs, $r_{\mathrm{UME}}$ is the radius of the UME, *D*_NP_ is the diffusion coefficient of the NP, which was estimated as 5.22 × 10^−7^ cm^2^ s^−1^ by the Einstein–Stokes equation. The *k*_ads_ was considered to be 1 for the theoretical expectation. The estimated *k*_ads_ based on the experimental frequencies were 2.6 × 10^−3^ and 1.4 × 10^−3^ for Au and Pt UMEs, respectively. The relatively larger value for the Au UME than for the Pt UME implied higher adhesion properties for the Pd NP on the Au UME.

The experimentally obtained collision frequencies were almost three orders of magnitude lower than the theoretical expectation. Differences between theoretical and obtained values could be explained by the presence of a varied adsorption behavior for each UME and other factors such as the aggregation of NPs.

It is worth noting that the diffusion of the Pd NPs on the UME does not account for all collision-related characteristics. As a result, any other component accounting for the surface state of the UME (and hence the adsorption coefficient) should be factored into the collision frequency prediction. Previous research has suggested that in the case of a Cu UME, the surface is not always in a stable condition due to the continual self-oxidation and subsequent reduction by hydrazine that might occur at certain voltages [31,32]. As a result, the Cu UME’s adsorption coefficient for the Pt NP can be reduced due to its unstable surface condition [20]. Even though our investigations did not cover the other adsorptive peculiarities that could be associated with the Ni, Au, or Pt UMEs, these peculiarities could potentially be some other nondiffusion-related reasons why there were some discrepancies between our theoretical and experimental values for the Ni, Au, or Pt UMEs. In addition, in an electrolyte solution, the NP can aggregate or adhere to the cell wall or other electrodes [33]. This loss of NP could be considerable and another reason for the reduced collision frequency.

As a result of the peak height and collision frequency analysis, the Pd NP did not seem to adhere well upon collision with the UME composed of Cu and Ni materials or maintain well its electrocatalytic activity even if it adhered. On the contrary, the Pd NP collided and stuck well with the Au and Pt electrodes, and their electrocatalytic properties were therefore well maintained on the electrode surface.

These results are meaningful because they can provide criteria for which material for the support electrode should be used in future applications of Pd NPs.

## 4. Conclusions

Pd NP was synthesized, and we investigated its single-NP collision with various UMEs, such as Cu, Ni, Au, and Pt UMEs, by an electrocatalytic amplification method. The current response of the Pd NP collision signal changed according to the material of the UME and the applied potential. For example, with the Au and Pt UMEs, as the applied potential increased, the collision signal changed from a staircase to a blip response. By analyzing the collision frequency and peak height, the adsorption between the Pd NP and each electrode material was predicted. The Pd NP seemed to collide and adhere better with the Au and Pt UMEs than with the Cu and Ni UMEs. In conclusion, for applications using the electrocatalytic properties of Pd NP, the use of a Au UME will be more effective than the use of Cu, Ni, and Pd UMEs as a supporting electrode.

## Figures and Tables

**Table 1:** Response types, peak heights, and frequencies of single-Pd-NPs collisions at Au and Pt UMEs obtained during the chronoamperometric measurements at 0.1 and 0.3 V.

UME	Response Type at 0.1 V	Response Type at 0.3 V	Peak Height ^[a]^ (pA) at 0.1 V	Frequency ^[b]^ (s^−1^ μm^−1^) at 0.1 V	Peak height ^[a]^ (pA) at 0.3 V	Frequency ^[b]^ (s^−1^ μm^−1^) at 0.3 V
Au	Staircase	Blip	700	0.051	625	0.050
Pt	Blip	Blip	325	0.028	416	0.071

^[a]^ The spike height of the blip responses at the Pt UME and current step of the staircase response at the Au UME. ^[b]^ Normalized value for the radius of each UME.

## Data Availability

Not applicable.

## Supplementary Materials

The following are available online at https://www.mdpi.com/article/10.3390/nano12183095/s1, Figure S1: The collision frequency and quality of single Pt NP collision at 5, 10, and 50 mM of PB (electrolyte), Figure S2: Chronoamperometric curves without the Pd NP at Cu, Ni, Au, and Pt UME with a 0.1 V (vs. Ag/AgCl) applied in a 50 mM phosphate buffer (pH 6.8) containing 15 mM hydrazine. The data acquisition time was 50 ms, Figure S3: Chronoamperometric curves without the Pd NP at Cu, Ni, Au, and Pt UME with a 0.3 V (vs. Ag/AgCl) applied in a 50 mM phosphate buffer (pH 6.8) containing 15 mM hydrazine. The data acquisition time was 50 ms, Figure S4: Chronoamperometric curves for a single Pd NP collision at Cu and Ni UME with a 0.1 V (vs. Ag/AgCl) applied in a 50 mM phosphate buffer (pH 6.8) containing 15 mM hydrazine. The Pd NP concentration was 160 pM. The data acquisition time was 50 ms, Figure S5: Chronoamperometric curves for a single Pd NP collision at Cu and Ni UME with a 0.3 V (vs. Ag/AgCl) applied in a 50 mM phosphate buffer (pH 6.8) containing 15 mM hydrazine. The Pd NP concentration was 160 pM. The data acquisition time was 50 ms.
