# Supplementary material for: Observation and Analysis of Staircase Response of Single Palladium Nanoparticle Collision on Gold Ultramicroelectrodes
Source: Nanomaterials (Basel). 2022 Sep 7;12(18):3095. doi: 10.3390/nano12183095 (PMC9500959; doi:10.3390/nano12183095)
Supplement: Supplementary file 1 [file nanomaterials-12-03095-s001.zip › nanomaterials-1845952-supplementary.pdf]

Supporting Information

Article

Observation and Analysis of Staircase Response of Single Palladium Nanoparticle Collision on Gold Ultramicroelectrodes

Hubert Rudakemwa†, Ki Jun Kim†, Tae Eun Park, Hyeryeon Son, Jaedo Na and Seong Jung Kwon*

Department of Chemistry, Konkuk University, 120 Neungdong-ro Gwangjin-gu Seoul 05029, South Korea
* Correspondence: sjkwon@konkuk.ac.kr; Tel.: +82-2-450-0429
† These authors are contributed equally.

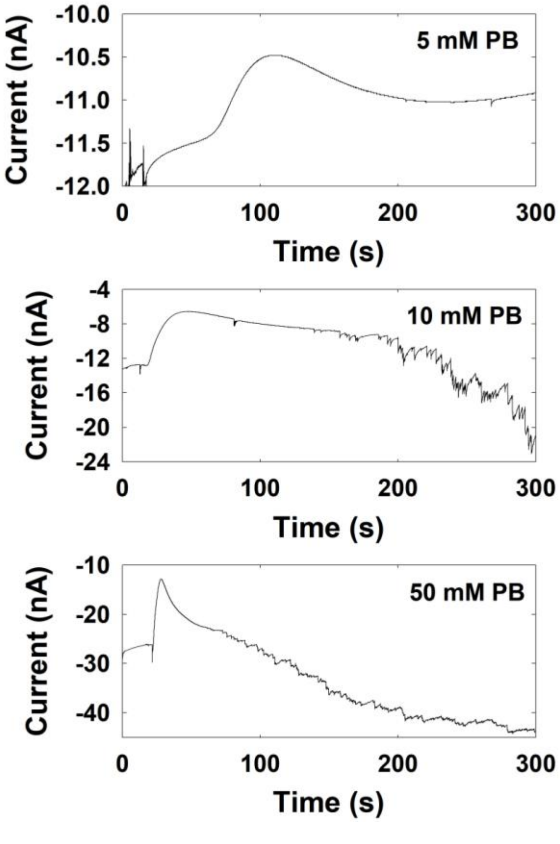

Figure S1. The collision frequency and quality of single Pt NP collision at 5, 10, and 50 mM of PB (electrolyte).

The 50 mM of electrolyte concentration is optimized for the best observation of current response. As shown here, the clear and frequency current response was observed at 50 mM concentration of electrolyte. At 5 and 10 mM concentration, the collisional frequencies are reduced much than the case of 50 mM. Therefore, we had optimized the concentration of electrolyte with 50 mM.

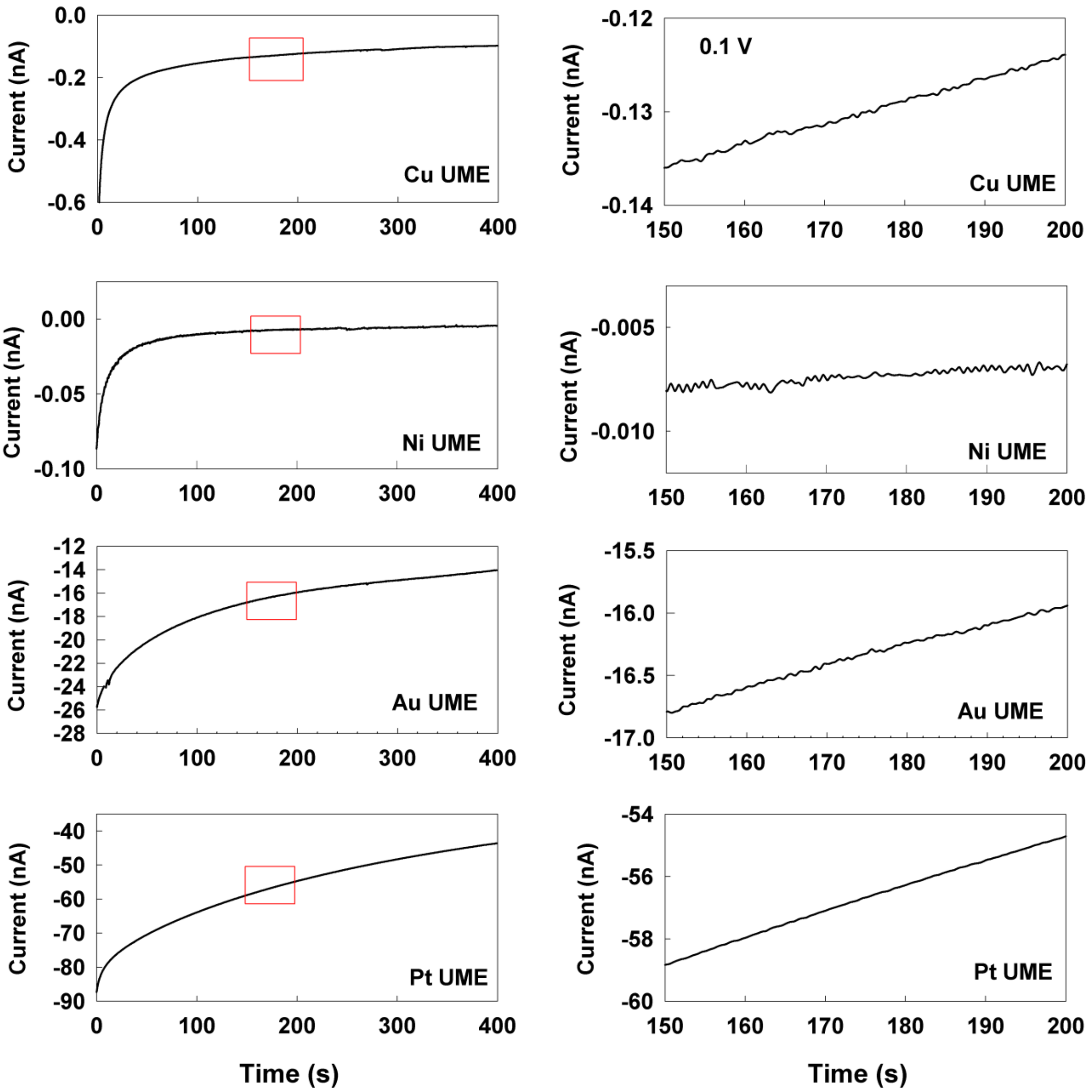

Figure S2. Chronoamperometric curves without the Pd NP at Cu, Ni, Au, and Pt UME with a 0.1 V (vs. Ag/AgCl) applied in a 50 mM phosphate buffer (pH 6.8) containing 15 mM hydrazine. The data acquisition time was 50 ms.

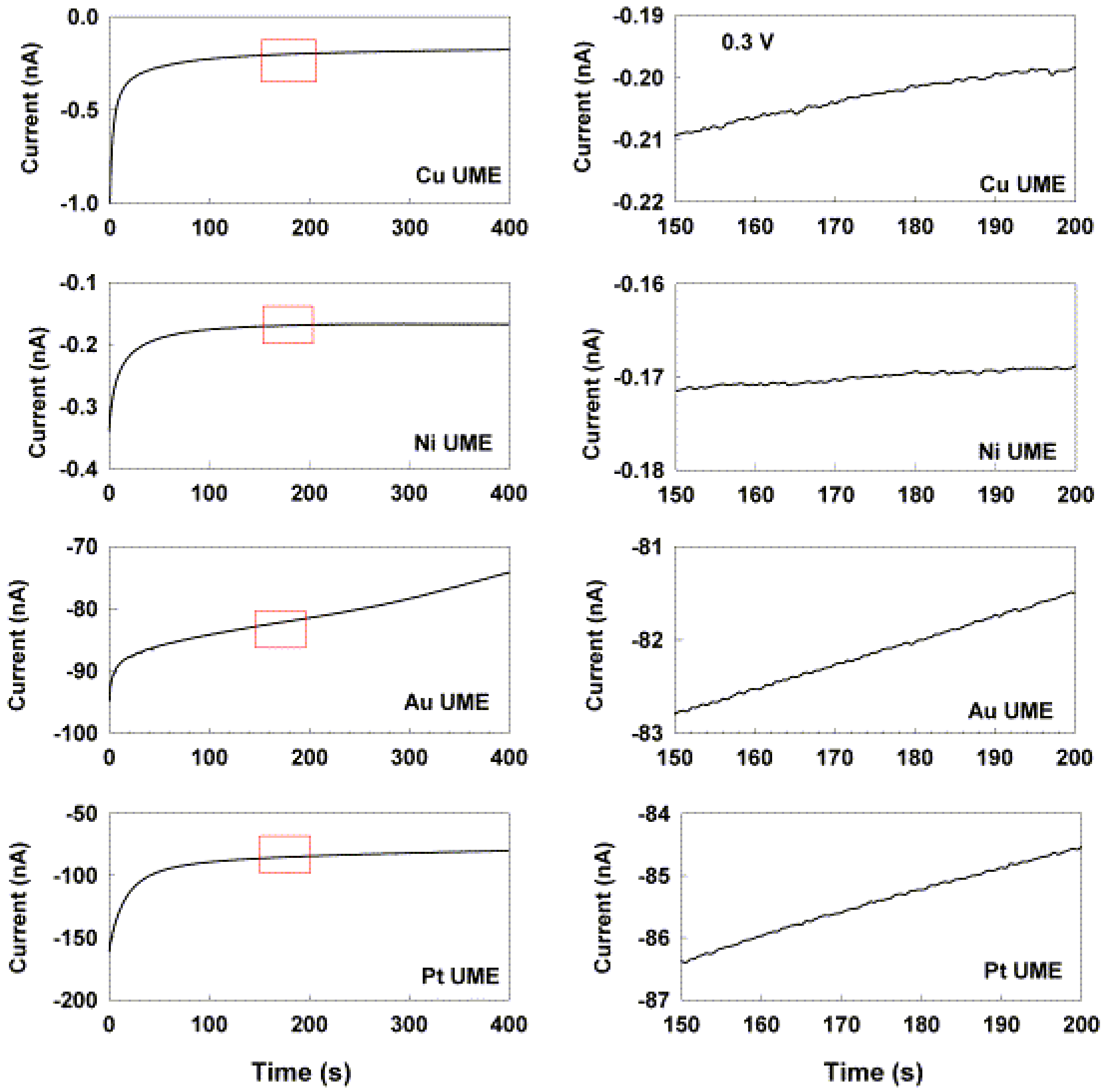

Figure S3. Chronoamperometric curves without the Pd NP at Cu, Ni, Au, and Pt UME with a 0.3 V (vs. Ag/AgCl) applied in a 50 mM phosphate buffer (pH 6.8) containing 15 mM hydrazine. The data acquisition time was 50 ms.

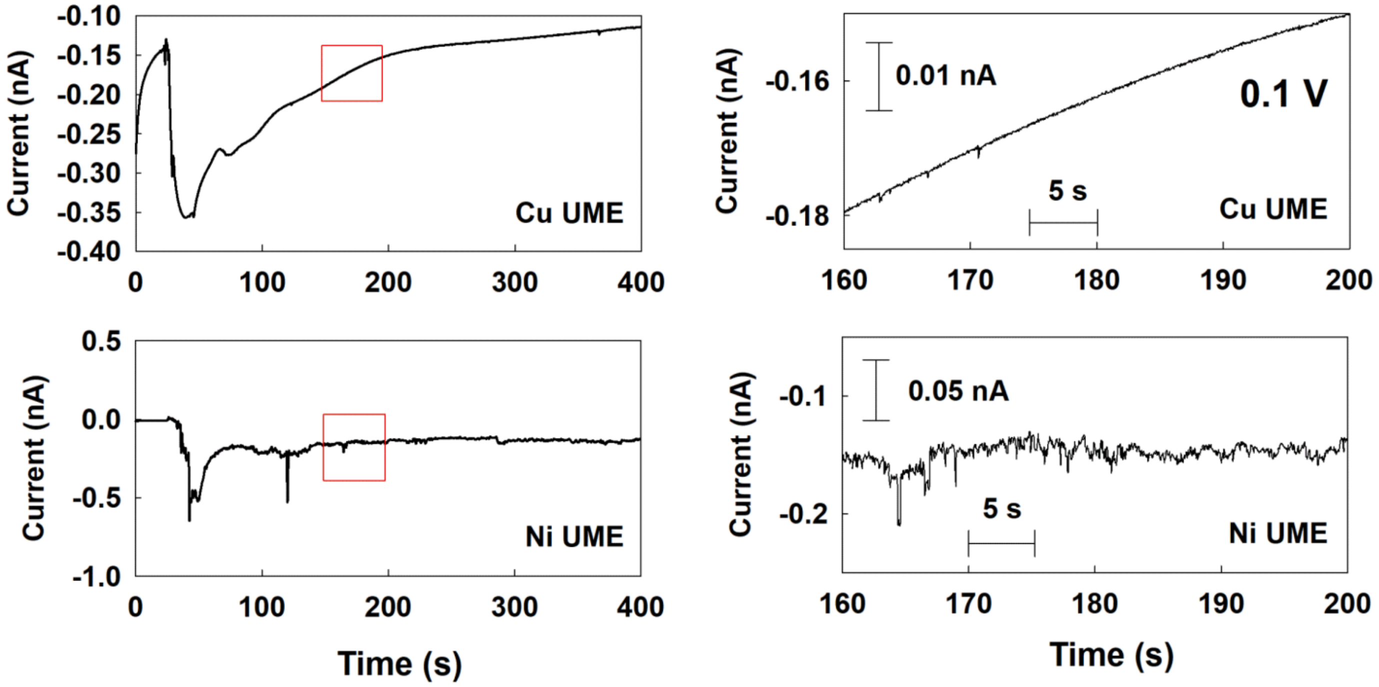

Figure S4. Chronoamperometric curves for a single Pd NP collision at Cu and Ni UME with a 0.1 V (vs. Ag/AgCl) applied in a 50 mM phosphate buffer (pH 6.8) containing 15 mM hydrazine. The Pd NP concentration was 160 pM. The data acquisition time was 50 ms.

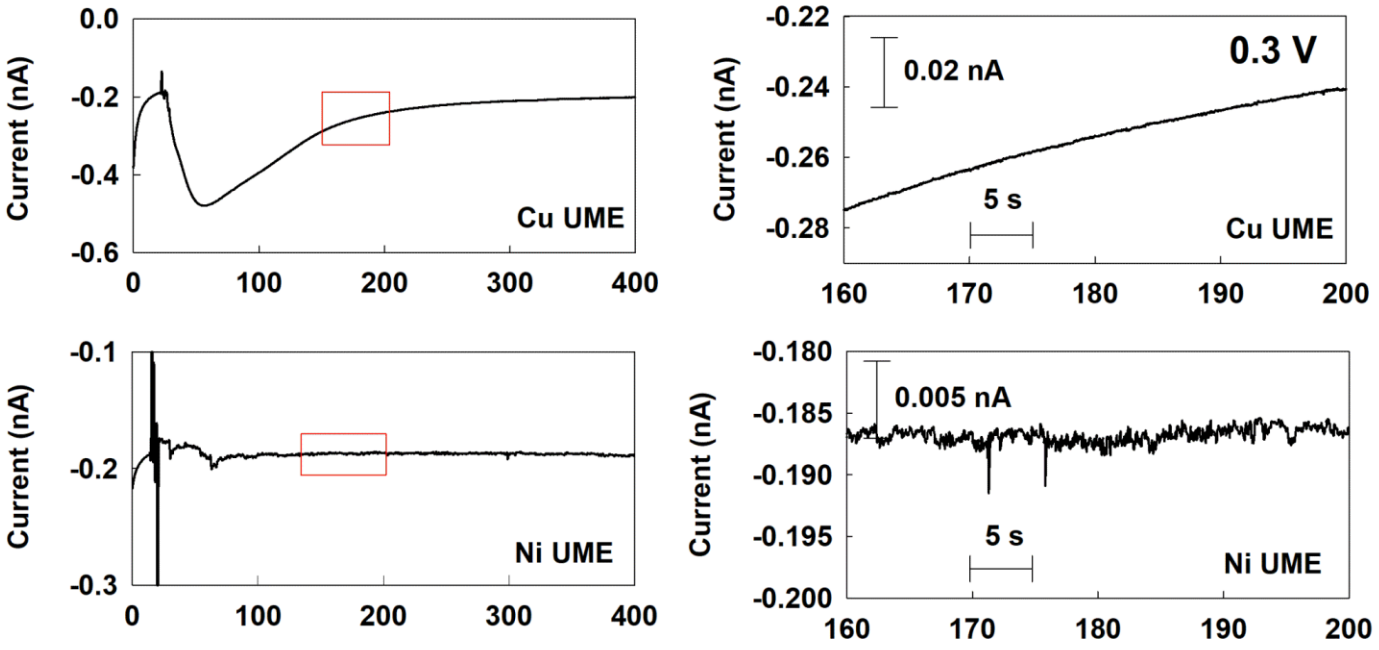

Figure S5. Chronoamperometric curves for a single Pd NP collision at Cu and Ni UME with a 0.3 V (vs. Ag/AgCl) applied in a 50 mM phosphate buffer (pH 6.8) containing 15 mM hydrazine. The Pd NP concentration was 160 pM. The data acquisition time was 50 ms.
